# Polygenic risk scores and early manifestations of attention‐deficit/hyperactivity disorder

**DOI:** 10.1002/jcv2.12103

**Published:** 2022-09-02

**Authors:** Henrik Larsson, Guilherme V. Polanczyk

**Affiliations:** ^1^ School of Medical Sciences Örebro University Örebro Sweden; ^2^ Department of Psychiatry Faculdade de Medicina FMUSP Universidade de São Paulo São Paulo Brazil

**Keywords:** early detection, early intervention, polygenic risk scores, preschool ADHD

A recent comprehensive systematic review and meta‐analysis explored the extent to which early neurocognitive and behavioural precursors are associated with the development of attention‐deficit/hyperactivity disorder (ADHD) and whether these are currently targeted in early interventions (Shephard et al., 2022). Understanding how ADHD emerge early in life and evolve is a very important research area as it has the potential to help improve early detection and identify new early intervention targets for ADHD (Sonuga‐Barke & Halperin, 2010). The systematic review by Shephard et al. (2022) identified a large number of studies (149 cross‐sectional or longitudinal studies) that together covered 8 early life neurocognitive and behavioural domains. Results from a series of multilevel random‐effects meta‐analyses suggested that pre‐school children with current or later‐emerging ADHD are likely to experience difficulties in multiple neurocognitive and behavioural functions.

In contrast to the large number of studies investigating early neurocognitive and behavioural precursors of ADHD, relatively few studies have assessed these as intervention targets. The systematic review by Shephard et al. (2022) included 28 randomized controlled trials (RCTs) and 4 nonrandomized trials testing a range of interventions that targeted relevant early precursors of ADHD. Multilevel random‐effects meta‐analyses of the included studies suggested that early interventions show some effectiveness in reducing ADHD symptoms and working memory, but their effects on other neurocognitive and behavioural difficulties remains unclear.

Several of the studies included in the above mentioned meta‐analyses used a cross‐sectional design. Shephard et al. (2022) therefore highlighted that longitudinal designs are needed to establish the predictive value of early neurocognitive and behavioural precursors as well as their potential causal role for ADHD. Another important focus for future research is to include biological markers, like genetic information, that can contribute to the predictive models (Farhat et al., 2020). A closer look at the included studies in the above mentioned systematic review and meta‐analysis reveals that few of the available studies incorporated genetic data in the study design. Such data could be used in prediction modeling research to develop tools for early detection. Genetic data could also be used to strengthen causal inference for observed association precursors/early markers and ADHD, which is a critical step towards identifying new early intervention targets for ADHD (Taylor, 2021).

## POLYGENIC RISK SCORES AND ADHD

Genome‐wide association studies (GWAS) have been instrumental for identifying common genetic variants across the genome associated with mental conditions and traits. Statistically significant findings have now emerged from GWAS for multiple traits, like educational attainment, and neurodevelopment disorders, like ADHD. For example, the latest GWAS findings from the ADHD Working Groups of the Psychiatric Genomics Consortium, identified 12 loci significantly associated with ADHD (Demontis et al., 2019). Using summery statistics from large‐scale GWAS it is possible to calculate individual polygenic risk scores (PRS; calculated by multiplying the number of risk alleles a person carries by the effect size of each variant, and then summing each of these products across all risk loci) in independent genotyped samples.

A recent systematic review explored how the ADHD PRS adds to our understanding of ADHD and associated traits (Ronald, de Bode, & Polderman, 2021). The study found that the ADHD PRS was associated with not only diagnosed ADHD but also with elevated ADHD trait scores, more externalizing behaviours, impaired working memory, lower educational attainment, reduced brain volume, higher BMI and reduced SES. The investigators also highlighted emerging evidence that the signal from the ADHD PRS remained significant after controlling for other PRSs, and that the ADHD PRS primarily appeared to associate with ADHD‐relevant phenotypes, which indicates some level of specificity in relation to the genetics of other mental health conditions. Even though the number of PRS studies have increased substantially in recent years, Ronald et al. (2021) and others (Larsson, 2021) have noted that there is still a lack of longitudinal ADHD PRS research. For example, more research is needed on how PRS associates with precursors and early markers of ADHD.

## POLYGENIC RISK SCORES AND EARLY MANIFESTATIONS OF ADHD

Two of the papers in the September issue of JCPP Advances represent useful examples of how the combined use of PRS and large‐scale, prospective studies data can be used to inform the field about early manifestations of ADHD and other neurodevelopmental conditions. The two publications have important strength such as the use of appropriate state‐of‐the‐art methodology, transparent reporting and use of a large representative sample and prospectively collected information using well‐validated instruments (i.e., ALSPAC).

First, the publication by Tobarra‐Sanchez et al. (2022) in the September issue of JCPP Advances explored the extent to which early markers and developmental characteristics during the first 30 months of life were associated with ADHD (i.e., ADHD trait scores and clinical diagnosis). Tobarra‐Sanchez et al. (2022) used data from 9201 participants from the prospective Avon Longitudinal Study of Parents and Children (ALSPAC) birth cohort. They identified 17 potential ADHD markers including pre‐ and peri‐natal risk factors, genetic liability (ADHD PRS), early development, temperament scores and regulatory problems. The main findings from this study indicated that high temperament activity levels and motor and speech delays in the first 30 months of life predicted ADHD 6 years later. Interestingly, given that PRS are known to explain little of the variance of ADHD outcomes, they found that ADHD PRS added useful predictive information over and above the other early markers and developmental characteristics.

Second, the study by Riglin et al. (2022) tested the hypothesis that measures of toddler language development, motor development and temperament are associated with genetic liability to neurodevelopmental disorders, including ADHD, autism and/or schizophrenia. Again, data were analysed from the ALSPAC cohort. Early measures included motor development scores at age 18 months and language development and temperament scores at age 24 months and genetic liability for neurodevelopmental disorders was indexed by PRS for ADHD, autism and schizophrenia. ADHD genetic liability was associated with better gross motor scores, higher activity and lower withdrawal, whereas schizophrenia genetic liability was associated with increased negative mood. They found no strong evidence of associations between autism genetic liability and early developmental characteristics.

## LIMITATIONS AND FUTURE DIRECTIONS

Tobarra‐Sanchez et al. (2022) and Riglin et al. (2022) discuss their main findings in context of three important limitations. These limitations are important to address in future research as they constrain a broader use of PRS in psychiatric research and clinical contexts.

First, PRS for ADHD and other neurodevelopmental conditions at present explain only a small fraction of variance in ADHD and early manifestations of ADHD, and is therefore a weak instrument. While larger GWAS samples will increase the predictive ability, ADHD PRS will likely never, on their own, be able to predict future diagnoses in a meaningful way. Nevertheless, it is possible that PRS in the future, through larger sample sizes and phenotypic refinement, could be used to improve outcome prediction and aid clinical decision‐making, together with other clinical variables. As suggested by Tobarra‐Sanchez et al. (2022), future research needs to explore whether predictive models incorporating aspects of early development and PRS are useful for predicting ADHD in clinical practice.

Second, because of a lack of ancestral diversity in currently available GWAS, individuals were excluded from the analyses on the basis of non‐European ancestry, which limits generalisability. Investments are needed to perform large‐scale GWASs in globally diverse populations. With more diverse samples, the genetic architecture of ADHD and other traits and conditions will be understood better globally and clinical applications of PRS will be more equally applicable across population groups.

Third, while PRSs are useful for studying correlations, they cannot be taken as evidence of causality. Riglin et al. (2022) correctly point out that they cannot differentiate whether motor development and temperament lie on the causal pathway between genetic liability to neurodevelopmental conditions and later neurodevelopmental conditions, if these associations arise independently as the result of (horizontal) pleiotropy, or whether the associations reflect an early manifestation of neurodevelopmental conditions. Implementation of sophisticated study designs and analyses will probably help uncover the mechanisms underlying identified PRS associations. As suggested by Riglin et al. (2022), research could investigate whether parental risk alleles for neurodevelopmental conditions (e.g., ADHD, autism and schizophrenia) are associated with differences in ratings of their children and whether un‐transmitted risk alleles using parent‐child trios contribute to “genetic nurture” effects. Mendelian Randomization methods can also complement findings from more standard PRS association analyses to strengthen causal inference, in particular when based on rigorous MR analyses with smaller numbers of highly significant variants using methods designed to account for pleiotropic bias.

## CONCLUSIONS

The two publications by Tobarra‐Sanchez et al. (2022) and Riglin et al. (2022) provides novel insight into associations between polygenic risk scores and early manifestations of ADHD. In the future, the combined use of aspects of early development, other relevant clinical information and PRS may be used to improve outcome prediction and aid clinical decision‐making. One important next step is to conduct rigor predicting modeling research (Senior et al., 2021) to test this hypothesis. More longitudinal studies using genetically sensitive designs are also needed to strengthen causal inference for observed association precursors/early markers and ADHD, as this is one important component in the work towards identifying novel early intervention targets for vulnerable infants and pre‐school children.

## AUTHOR CONTRIBUTIONS

Henrik Larsson: Conceptualization; Writing – original draft; Writing – review & editing. Guilherme V. Polanczyk: Writing – original draft; Writing – review & editing.

## CONFLICTS OF INTEREST

Henrik Larsson reports receiving grants from Shire Pharmaceuticals; personal fees from and serving as a speaker for Medice, Shire/Takeda Pharmaceuticals and Evolan Pharma AB; and sponsorship for a conference on attention‐deficit/hyperactivity disorder from Shire/Takeda Pharmaceuticals and Evolan Pharma AB, all outside the submitted work. Henrik Larsson is Editor‐in‐Chief of JCPP Advances. Guilherme, Guilherme V. Polanczyk reports receiving personal fees from and serving as a speaker and/or consultant for Abbott, Ache, Medice, Novo Nordisk, Takeda and royalties from Editora Manole. Guilherme V. Polanczyk is a Joint Editor for JCPP Advances.
